# Evolution of facial color pattern complexity in lemurs

**DOI:** 10.1038/s41598-017-15393-7

**Published:** 2017-11-09

**Authors:** Hanitriniaina Rakotonirina, Peter M. Kappeler, Claudia Fichtel

**Affiliations:** 1 Behavioral Ecology & Sociobiology Unit, German Primate Center, Göttingen, Germany; 2 Wissenschaftskolleg zu Berlin, Wallotstr. 19, 14193 Berlin, Germany

## Abstract

Interspecific variation in facial color patterns across New and Old World primates has been linked to species recognition and group size. Because group size has opposite effects on interspecific variation in facial color patterns in these two radiations, a study of the third large primate radiation may shed light on convergences and divergences in this context. We therefore compiled published social and ecological data and analyzed facial photographs of 65 lemur species to categorize variation in hair length, hair and skin coloration as well as color brightness. Phylogenetically controlled analyses revealed that group size and the number of sympatric species did not influence the evolution of facial color complexity in lemurs. Climatic factors, however, influenced facial color complexity, pigmentation and hair length in a few facial regions. Hair length in two facial regions was also correlated with group size and may facilitate individual recognition. Since phylogenetic signals were moderate to high for most models, genetic drift may have also played a role in the evolution of facial color patterns of lemurs. In conclusion, social factors seem to have played only a subordinate role in the evolution of facial color complexity in lemurs, and, more generally, group size appears to have no systematic functional effect on facial color complexity across all primates.

## Introduction

Fur, skin and plumage coloration are highly diverse in animals^1–3^ and can take on many functions, such as communication^4–9^, thermoregulation^5,8–10^, or predation avoidance^11–14^. For example, plumage coloration in birds can provide camouflage (e.g. in ruffed grouse, *Bonasa umbellus*)^15,16^ or information used in mate choice (e.g. in cattle egrets, *Bubulcus ibis ibis*)^17^. Chameleons use their coloration for background matching to avoid detection by predators (e.g. in dwarf chameleons, *Bradypodion taeniabronchum*)^18,19^. In mammals, variation in pelage coloration can also reduce detection by predators (e.g. in oldfield deer mice, *Peromyscus polionotus*)^20^, support thermoregulation (e.g. the dark pelage of tropical bovids)^21^, or serve as a signal in social communication (e.g. the facial color pattern in New World monkeys)^8^ and species recognition (e.g. the face patterns in guenons)^22^. Accordingly, a combination of social and ecological selective pressures has driven the enormous variation in animal coloration^1,21,23,24^.

Among mammals, primates exhibit remarkable variation in skin and pelage coloration^25,26^, perhaps because they exhibit more variation in ecology, activity period and social systems than the other three large orders (rodents, bats and shrews), which together make up more than 70% of all mammals^27^. Because of this variation, in combination with the presence of trichromatic vision in the majority of species, primates are an excellent group for elucidating factors influencing variation in coloration within and among species^1,25,28^. Previous studies revealed that intra- or interspecific variation in facial hair or skin color as well as hair length in primates may have evolved in response to social and ecological pressures^8,9,22,29^. Whereas intraspecific variation in facial coloration has been suggested to play a role in social interactions because it contains information about an individual’s identity, status and condition^8,9,29–35^, interspecific variation has been suggested to reflect social and ecological adaptations. For example, in Neotropical primates, interspecific variation in facial color pattern was greater in species living in smaller groups and explained by the possibility of greater reliance on facial expression in species living in larger groups^8^. However, in Old World primates, the opposite patterns were found, with species living in larger groups exhibiting more variation in facial color patterns^9^. Interspecific variation in facial color pattern as found in New and Old World monkeys has also been linked to the need for reliable species recognition. Species living with more sympatric congeners evolved indeed more complex facial color patterns than species living without or with fewer sympatric congeners, possibly to reduce the risk of hybridization^8,9,22^_._

Variation in facial hair length and color has also been linked to ecological factors. For example, both New and Old World primates with longer facial hair occur more often in cooler areas, and those exhibiting darker facial areas tend to occur more often in denser forests rather than in more open habitats^8,9^. Moreover, species occurring closer towards the equator sport darker coloration in some regions of the face (crown and eye mask), lighter coloration in others (nose and mouth), and they have shorter facial hair^8^. Hence, facial color pattern complexity and variation in Old World and New World primates exhibit some convergent patterns, but they also diverge from each other in response to some social selective factors, but functional explanations for these associations are not available as yet.

**Figure 1:**
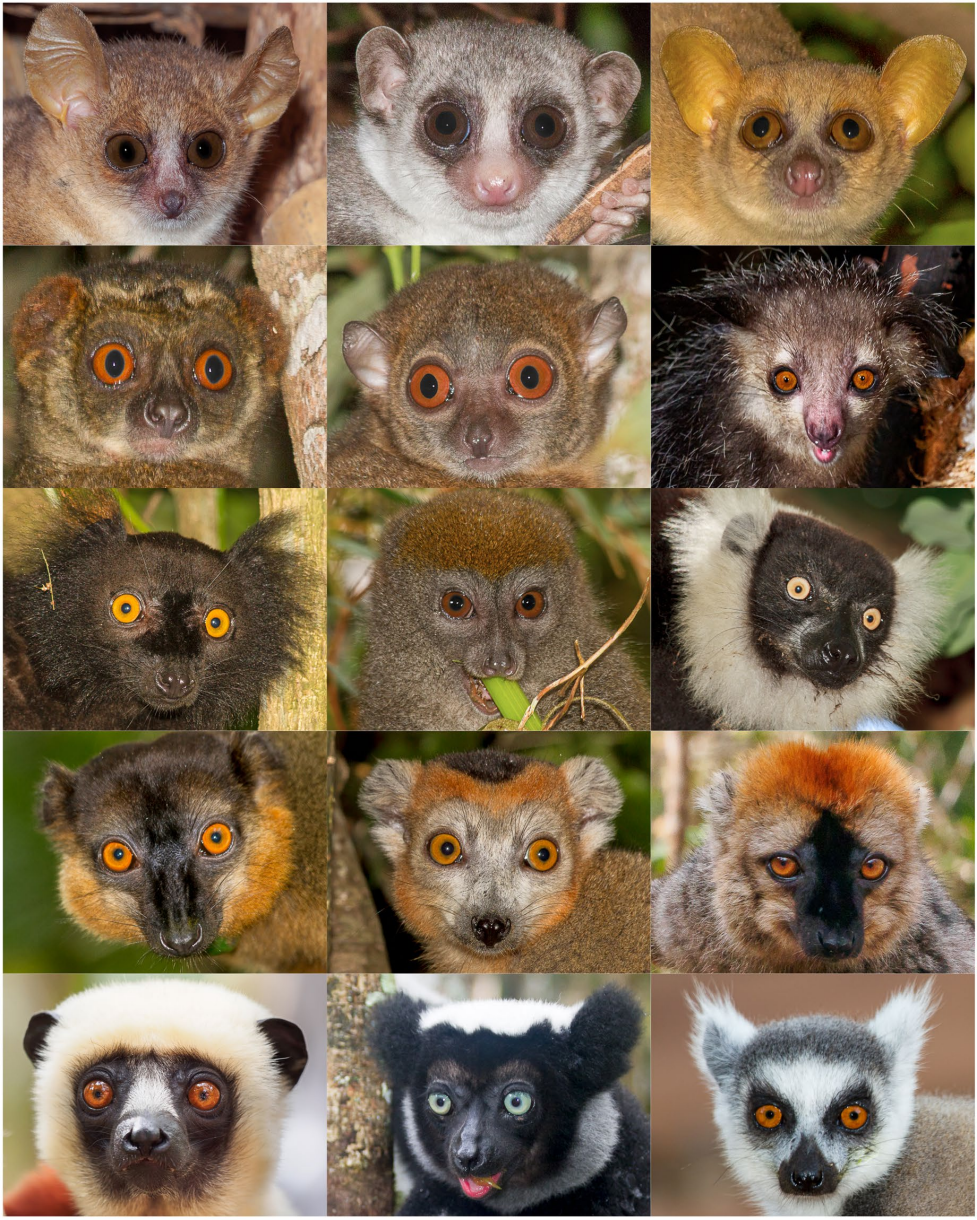
Examples of facial color patterns diversity in lemurs. Presented are (left to right): nocturnal species including *Microcebus murinus*, *Cheirogaleus medius*, *Mirza coquereli*, *Avahi laniger*, *Lepilemur dorsalis*, *Daubentonia madagascariensis*, cathemeral species: *Eulemur macaco*, *Hapalemur griseus*, *Varecia variegata*, *Eulemur collaris, Eulemur coronatus, Eulemur rufifrons*; diurnal species: *Propithecus coquereli*, *Indri indri*, *Lemur catta*. (All photographs taken by M. Markolf).

The adaptive radiation of primates endemic to Madagascar (Lemuriformes) provides an opportunity for an independent test of these relationships because they evolved in isolation from other primates for more than 50 million years^36^. With currently more than 100 recognized species, lemurs are taxonomically diverse, they occupy a range of different forest habitats from dry to humid forests^37,38^, and they exhibit all major forms of social organization found among anthropoid primates^39,40^. They also exhibit variation in activity patterns, including nocturnal, cathemeral and diurnal species^41^. Only members of two lemur genera, the true lemurs (*Eulemur*) and some mouse lemurs (*Microcebus spp*.), occur in sympatry with a maximum of one congeneric species per location, whereas most of the other species live in sympatry with at least one species belonging to the same family^36^. Hybridization risk is presumably higher for closely-related species from the same genus than for more distantly-related ones belonging to the same family. Most importantly, lemurs exhibit spectacular diversity in pelage coloration, particularly, in facial color patterns across families and species (Fig. 1). Diurnal or cathemeral lemur species have dichromatic vision, but some females exhibit polymorphic trichromacy, allowing them to perceive red and orange colors^25,42–44^. Although some nocturnal species lack dichromatic color vision^45^, differences in the brightness or contrasts of facial patches might be conspicuous for them. Thus, from the perception side, variation in color patterns should be meaningful for lemurs, although this assumption remains to be demonstrated experimentally.

The aim of this study was to investigate factors shaping facial color and hair patterns in lemurs. Specifically, because group size had opposite effects on interspecific variation in facial color patterns in the two radiations of New and Old World monkeys^8,9^, a study of the third large primate radiation may shed light on convergences and divergences in this context. Based on the results of these previous studies, we predicted that variation in facial color patterns in lemurs should be related to group size, and that variation in facial coloration should increase with the number of sympatric species. Furthermore, facial hair should be longer in lemurs inhabiting colder areas, and hair and skin coloration in different facial regions should be darker in species occurring in dense forest habitats.

## Results

### Facial pattern complexity, sociality and species recognition

**Figure 2:**
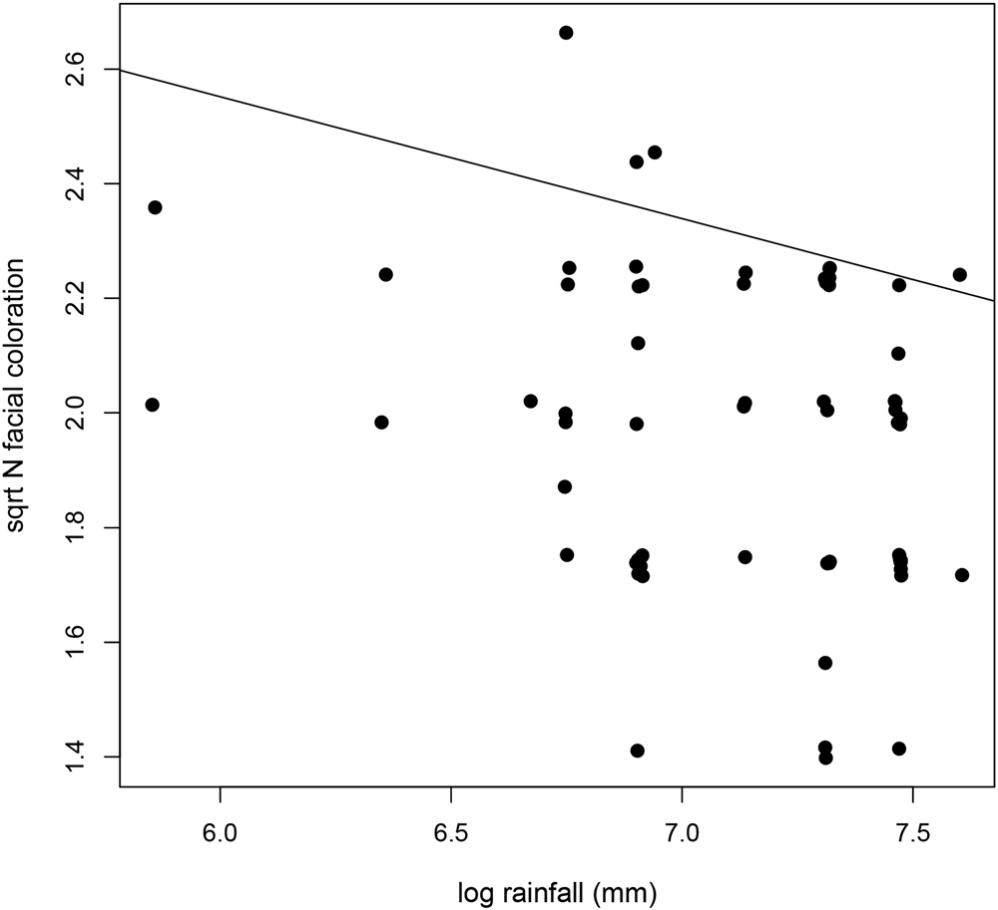
The relationship between rainfall and facial color complexity in lemurs. Facial color complexity is significantly lower in species inhabiting areas with higher rainfall.

We conducted Phylogenetic Generalized Least Square (PGLS) regressions to test for evolutionary relationships among diversity in facial color patterns and social as well as ecological variables. PGLS also estimates phylogenetic signal (here Pagel’s lambda^46^), which is the tendency for closely related species to resemble each other more in a certain trait value due to their phylogenetic proximity. Pagel’s lambda can range from zero to one. A lambda of zero indicates no phylogenetic signal and that the trait evolved independent of phylogeny. A lambda of one means strong phylogenetic signal and the trait evolved according to the phylogenetic tree^47^. Facial color complexity was independent of the number of sympatric species at the family level and group size in all models, independent of whether temperature, elevation range or rainfall were included as climate variables (Table S1). Rainfall was linked to facial color complexity, with species living in areas with lower rainfall exhibiting higher average facial complexity (PGLS: = 0.7, Estimate = −0.22, SE = 0.09 and p = 0.015; N = 65; Fig. 2; Table S1). However, this relationship might be driven by a few data points (from *Lepilemur hubbardorum, L. leucopus, Microcebus griseorufus and M. berthae*, which occur in some of the driest habitats).

### Facial regions: pigmentation, hair length and ecology

#### Crown

Hair color and hair length were neither correlated with any of the climate variables, nor the number of sympatric species on the family level, nor by group size (Table S2).

#### Forehead

**Figure 3:**
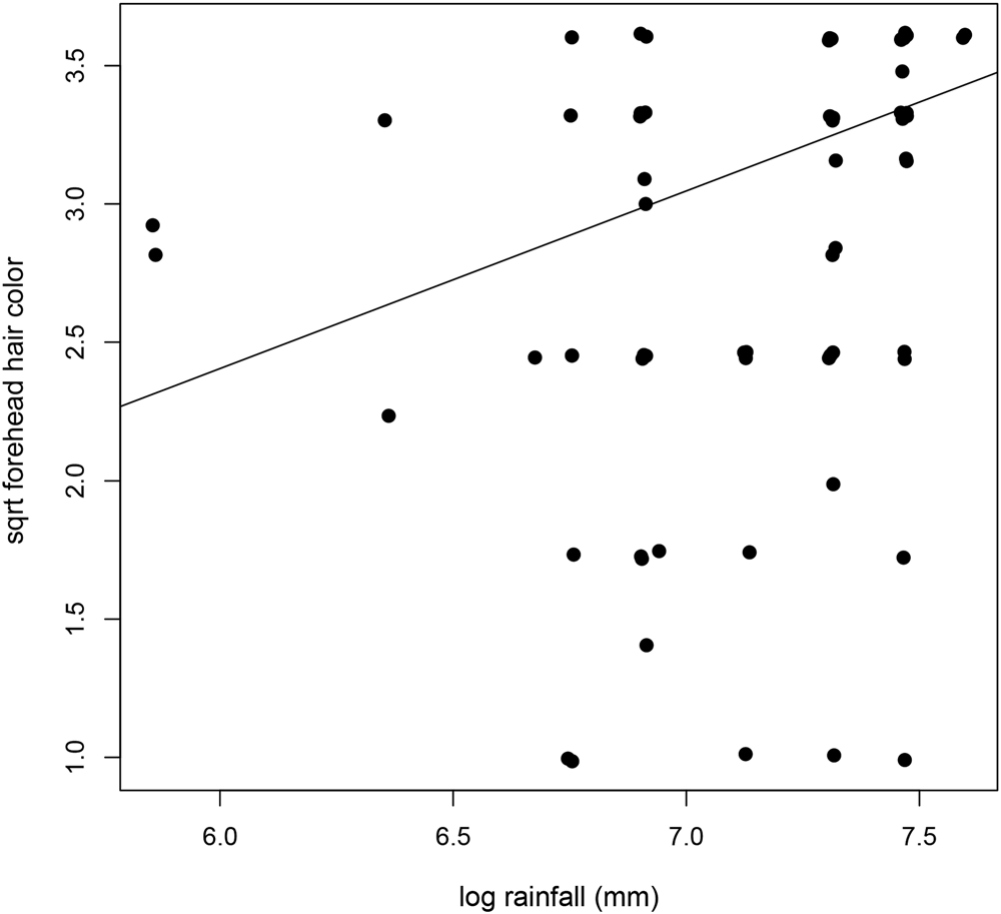
The relationship between rainfall and the color of the forehead. Species inhabiting areas with higher rainfall have a significantly darker forehead.

Hair color and length were not correlated with temperature or elevation range, number of sympatric species or group size (Table S3). However, rainfall was associated with hair color, with species occurring in habitats with higher rainfall exhibiting on average a darker forehead (PGLS: = 0, Estimate = 0.64, SE = 0.29 and p = 0.032, N = 65; Fig. 3, Table S3). These effects were also driven by the same four species occurring in very dry habitats.

#### Eyes

**Figure 4:**
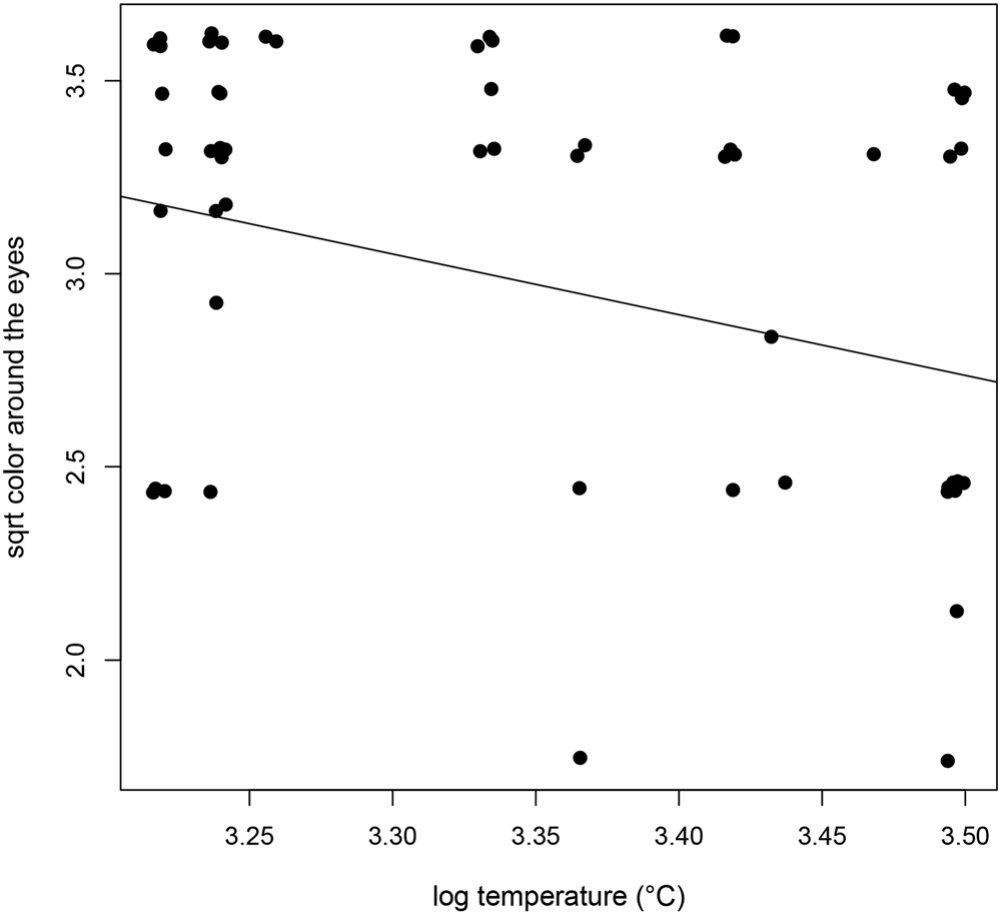
The relationship between temperature and hair color around the eyes. Species inhabiting cooler areas have significantly darker periocular hair.

The pigmentation or hair color in the peri-ocular region co-varied with temperature (PGLS: =0.52, Estimate = −1.57, SE = 0.5 and p = 0.003, N = 65) and rainfall (PGLS: = 0.48, Estimate = 0.4, SE = 0.16 and p = 0.014, N = 65), but not with elevation range, the number of sympatric species on the family level and group size (Table S4). Species occurring in colder habitats with more rainfall exhibited darker coloration in the area around the eyes (Fig. 4). Hair length varied independently of any of the analyzed predictor variables (Table S4).

#### Face margins

**Figure 5:**
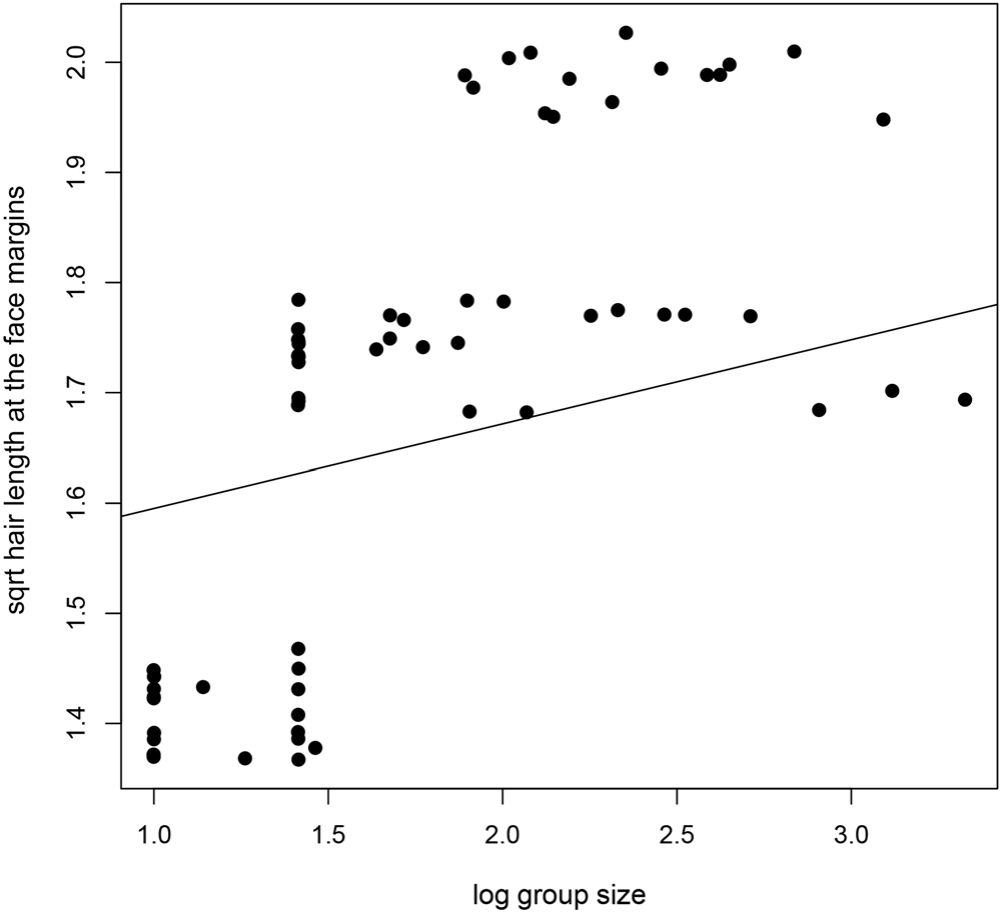
The relationship between lemur group size and hair length in the face margins. Species living in larger groups have longer hair in the face margins.

Hair color and length were not associated with temperature, elevation range, or the number of sympatric species (Table S5). However, rainfall correlated with hair color (PGLS: = 0, Estimate = 0.62, SE = 0.27 and p = 0.024, N = 65), but not length, with species occurring in habitats with higher rainfall exhibiting darker face margins. Hair length was correlated with group size in all models, with species living in larger groups having longer hair (Fig. 5, Table S5).

#### Ears

Hair color was not associated with any climate variable, the number of sympatric species or group size (Table S6). However, skin color varied with group size, with species living in larger groups having on average darker skin (Table S6). Hair length correlated with temperature (PGLS: = 0.75, Estimate = −0.88, SE = 0.27 and p = 0.002, N = 65), elevation range (PGLS: = 0.79, Estimate = 0.19, SE=0.07 and p = 0.007, N = 65), rainfall (PGLS: = 0.77, Estimate = 0.19, SE = 0.09 and p = 0.049, N = 65) and group size (Table S6). Species living in colder, higher and wetter habitats as well as those living in larger groups, had longer hair on the ears.

#### Skin color nose

The skin color of the nose did not co-vary with any of the predictor variables (Table S7).

Overall, ecological variables were associated with some color variation in facial regions and hair length on the ears in lemurs. In addition, the length of the hair of the face margins and ears co-varied with group size. All models showed strong phylogenetic signal.

## Discussion

This study investigated the influence of social and ecological variables on the diversity of facial color pattern in lemurs. Facial color pattern complexity in lemurs could not be explained by social variables. Neither the number of sympatric species nor group size influenced facial color pattern complexity. However, ecological variables explained some of the variation: species living in habitats with less rainfall had more complex facial color patterns. In addition, species living in habitats with more rainfall were darker around the eyes, had darker hair on the forehead and in the face margins. Similar, species occurring in colder, wetter and higher habitats had longer hair in the face margins and on the ears and lived in larger groups. We also found a moderate to high phylogenetic signal for most models, suggesting an impact of phylogeny on the evolution of facial color patterns in lemurs. Below, we first discuss overall color pattern complexity before focusing on the different facial regions.

### Facial color pattern complexity

Since heterospecific mating is costly, species recognition may serve as a premating isolation mechanism to avoid costly hybridization^1,22,29,48–51^. Accordingly, it has been proposed that interspecific diversity in facial color patterns in New World and Old World primates has evolved in the context of species recognition, with species occurring with more sympatric heterospecifics evolving more complex facial patterns^8,9,29^. In guenons, the faces of sympatric species have become more distinctive through character displacement^22^. In lemurs in contrast, only some *Eulemur ssp*. and *Microcebus ssp*. occur in sympatry with only one sympatric congener each, and the number of sympatric species belonging to the same taxonomic family did not influence facial color complexity. Since the risk of heterospecific mating is likely higher among congeners, and most lemurs occur in allopatry with their congeners, there might be only a weak selective pressure on facial color complexity as cues for reliable species recognition in this radiation of primates. However, lemurs can use variation in facial color patterns to discriminate between species, with brown (*E. fulvus*) and black lemurs (*E. macaco*) discriminating between pictures of their own species and heterospecifics (familiar and unfamiliar con- and heterospecifics)^52^. Thus, although this study suggests that facial color patterns in lemurs might not have evolved in the context of species recognition, this does not necessarily mean that cues of facial color patterns cannot be used secondarily for species recognition.

Facial color patterns have also been linked to group size, with New World monkeys living in smaller groups exhibiting more complex facial color pattern, whereas Old World monkeys exhibit the opposite effect^8,9^. In lemurs, group size did not co-vary with facial color pattern complexity. Lemurs differ from other primates in modal social organization and group size, however. Most lemur species are solitary or pair-living^53^, and group-living lemurs live in much smaller groups^39,40,53,54^ than colobines^55^, cercopithecines^56^ and apes^57^. These broad differences in social systems among the different primate radiations may explain why group size does not predict lemur facial color complexity. From a comparative perspective, it is therefore questionable whether group size has a systematic, general functional effect on primate facial color complexity. But how can the different patterns observed in the three independent primate radiations then be explained?

We propose that, instead of group size, complexity in facial color patterns might be related to facial mobility in primates, i.e. their ability for facial expression^58^. Indeed, species with lower facial mobility or less expressive faces have a higher facial complexity^58^. It has been argued that complex facial color patterns potentially allow greater intraspecific and interspecific variation, facilitating recognition at the level of individuals or species^8,9^. Because lemurs have reduced facial mobility and ability for facial expressions among the species investigated so far (e.g. in geladas^59^, rhesus monkeys^60^, chimpanzees^61^, ring-tailed lemurs^62^), they should show the greatest facial color complexity according to this notion.

A direct comparison between facial color complexity among published studies is difficult as the number of facial areas and color categories varied across studies, but a comparison of mean facial color complexity of catarrhine and strepsirrhine families indicated indeed higher facial color complexity in lemurs (Cheirogaleidae = 4.91, Daubentoniidae = 5.30, Indriidae = 3.20, Lemuridae = 4.16, Lepilemuridae = 3.33; Catarrhines^9^: Cercopithecinae = 3.54, Colobinae = 2.60, Hominidae = 1.50, Hylobatidae = 2.21). Hence, facial color complexity might indeed be related to the ability for facial expressions^58^. Therefore, future studies should aim at examining whether intraspecific variation in facial color patterns are used as cues for individual recognition (cf.^34^).

However, part of the variation in facial color complexity might also be driven by other factors.First, lemurs living in habitats with higher rainfall exhibited more complex facial color patterns. Since rainforests are usually characterized by lower visibility, more complex facial patterns may facilitate inter- and intraspecific recognition in this habitat type. Second, we could not investigate the potential roles of pathogen resistance or mate choice in this study, but there are indications that they may also impact the evolution of facial color complexity in primates^1,61–63^. Finally, we found moderate to high values of Pagel’s lambda^46^ in almost all models, indicating a strong effect of phylogeny on the diversification of lemur facial patterns. Hence, genetic processes such as genetic drift, also need to be invoked to fully explain the evolution of facial color complexity in lemurs.

### Variation in different facial regions

It has been suggested that environmental factors, such as habitat type or climate, can affect fur coloration and hair length in mammals^1,21,66^. Coat color across primates, for example, has evolved under Gloger’s rule^67^, where darker pelage colors are found more often in species occurring in forested habitats^68^. Hair length varies according to the “hair rule”^69^, with species living in colder areas having longer and thicker hair^8,9^. Our results on lemurs partly support both rules. Although we found no correlation between climate and hair color or length on the crown, climatic factors were associated with darker hair colors on the forehead and face margins as well as with increased pigmentation around the eyes. Additionally, species living in colder, higher and wetter areas of Madagascar tend to have longer hair on the ears.

Madagascar’s climate is highly diverse^70,71^, and it has been suggested that warmer regions on the island are characteristic of more open and dry forests, and lower elevations^37,72,73^. Thus, in support of Gloger’s rule, lemur species in eastern, colder and more forested habitats developed darker hair on the forehead and the face margins and darker color around the eyes, and longer hair on the ears following the hair rule^69^. However, pigmentation and hair length of the other facial areas might have evolved due to genetic drift or other selective pressures not investigated in this study. For example, selective pressures related to predator avoidance, such as crypsis, countershading, disruptive coloration or background matching may have played a role in the evolution of pelage coloration of the body^1,25,74^. Interestingly, the hair length of the face margins and ears was also correlated with group size, with species living in larger groups having longer hair. Thus, intraspecific variation in hair length might be used to discriminate conspecifics, but neither the data quantifying intra-specific variation in these traits nor the corresponding experiments with visual stimuli are currently available to examine this notion.

## Conclusions

This is the first study investigating the evolution of facial pattern complexity in the independent adaptive radiation of lemurs, the endemic primates of Madagascar. We found that lemur facial variation was not influenced by social variables and only weakly by climatic and other ecological factors. Comparative analyses indicated a notable influence of phylogeny on the evolution of lemur facial patterns, perhaps reflecting the relatively recent and rapid divergence of lemurs. Moreover, and in contrast with previous studies of New and Old World monkeys, group size was unrelated to facial color complexity in primates. Thus, the evolution of facial color and hair patterns in primates is characterized by little convergence, suggesting that lineage-specific factors need to be considered in comprehensive analyses as well.

## Materials and Methods

### Variation in facial patterns

To investigate facial pattern complexity in lemurs, photographs from private collections, photographers and the Internet (All the World’s Primates (http://www.alltheworldsprimates.org) and Arkive (http://www.arkive.org)) were chosen in order to quantify and categorize hair and skin color variation as well as hair length. Two to ten photographs with high resolution per species were chosen and categorized using Adobe Photoshop CS3 with the highest brightness of the screen. Each picture contained the photograph of one individual looking towards the camera where all areas of the face were well visible. Only photographs of adults were taken and categorized (Fig. 1). We only used photographs of adult males from *Eulemur* species because they exhibit sexual dichromatism, with males being the more colorful sex. We collected data from 65 lemur species and analyzed 522 photographs.

#### Categorization of hair and skin color variation

**Figure 6:**
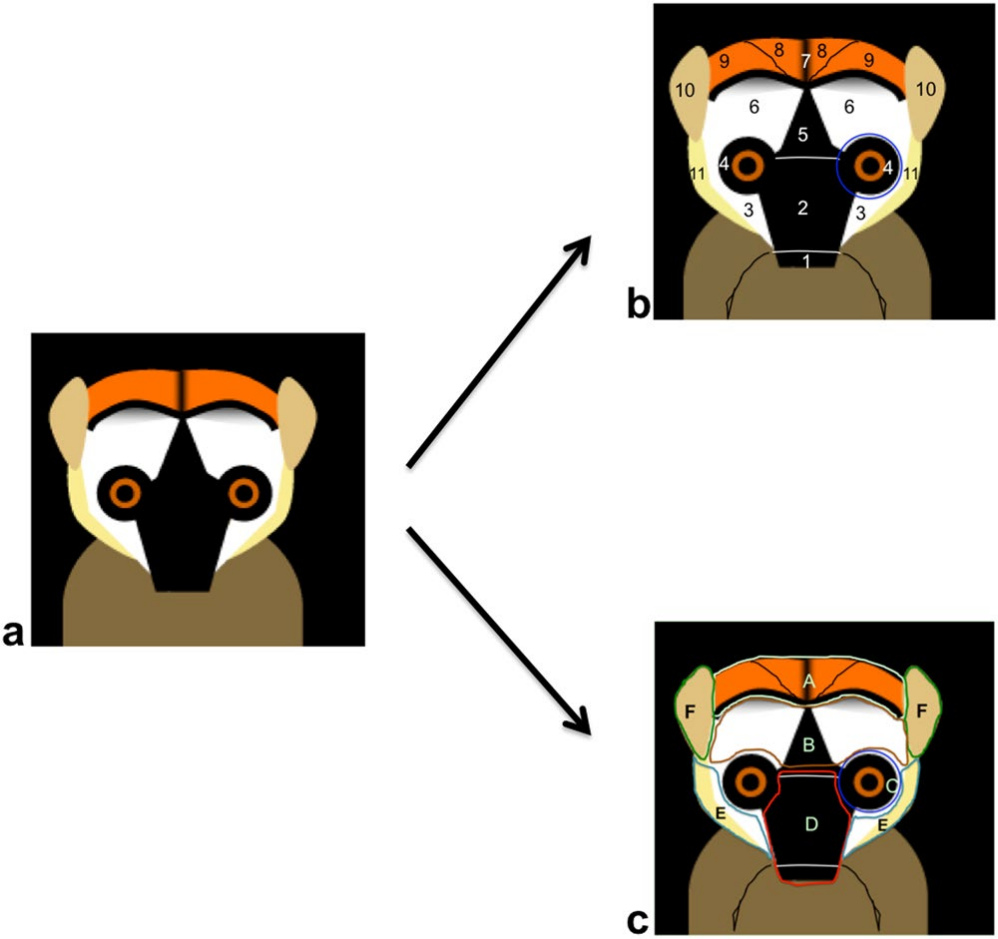
Schematic face (**a**) used to divide lemur faces into different areas (**b**) and regions (**c**) used to assess hair and skin color as well as hair length variation (modified after Barthold *et al*.^77^). Schematic face of a lemur. (**b**) Face of a lemur divided in 11 areas: 1 = nose; 2 = area above the nose; 3 = area below the eyes; 4 = eye contour (in blue); 5 = center area above the eyes; 6 = forehead; 7 = center of the crown; 8 = first area of the crown; 9 = second area around the crown; 10 = ears; 11 = face margins. (**c**) Face of the lemur divided in 6 regions: A = crown (areas 7 + 8 + 9); B = forehead (areas 5 + 6); C = eyes (area 4); D = nose and mouth (areas 1 + 2); E = face margins (areas 3 + 11); F = ears (area 10). All the areas are here combined into regions.

Our categorization followed the procedures described by Santana *et al*.^8,9^, who examined facial hair color, skin color and hair length to investigate facial pattern complexity. In order to categorize facial coloration in lemurs, each photograph was first divided into 11 areas (Fig. 6). For each area, we determined hair coloration (classified as either white, agouti, brown, grey or yellow, Fig. S1) and skin coloration (classified as depigmented (no hair), pigmented (pink skin, mottled, or gold skin) to hyper-pigmented (black or brown), Fig. S2). For hair coloration, each color was additionally classified as light, medium or dark, depending on the intensity of pigmentation (Fig. S1). Furthermore, in order to determine the intensity of brightness (from light to dark) in facial hair, we divided the face into 6 regions (crown, forehead, eyes, ears, mouth and face margins, (Fig. 6)), and we determined the most predominant color (~90% of all color) in each region of the face. Additionally, we categorized hair length per region (classified as either depilated (or no hair), short, medium to long, Fig. 6, Fig. S3).

#### Ecological and social variables

We collected data on Malagasy ecoregions and habitats based on their distributions and a classification made by Muldoon and Goodman^37^. Ecoregions were classified as follows: spiny thicket, succulent woodland, dry deciduous forest, subhumid forest and humid forest^37^. We also determined the maximum temperature (in following: temperature), upper elevation range (elevation range), the minimum rainfall (rainfall) and for each ecoregion where a species occurs and calculated an average value if a given species occurred in different ecoregions. As social factors, we collected data on social organizations (solitary, pair living or group living), on average group size and activity patterns (nocturnal, cathemeral or diurnal activities) for each species (Table S8). Additionally, we determined the number of sympatric species for each species in each ecoregion and determined the total number of sympatric species at the family level (and genus level, Table S9) based on the species compilation in Muldoon & Goodman^36^.

### Statistical analyses

#### Phylogenetic tree

**Figure 7:**
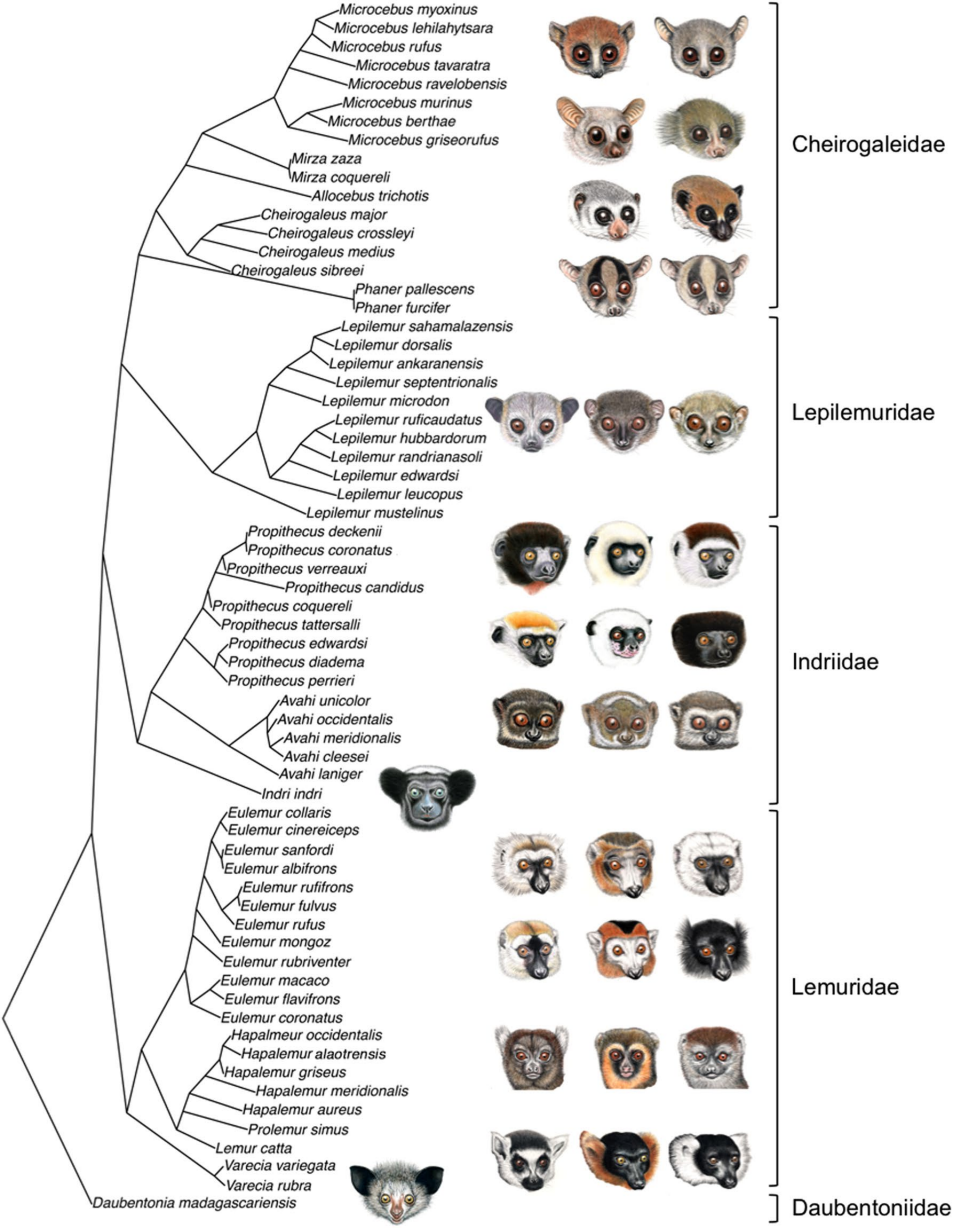
Phylogenetic tree of all lemur species used for Phylogenetic comparative analyses in this study (illustrations: S. Nash).

For a comparative phylogenetic analysis of variation in facial patterns, we obtained a phylogenetic tree of the species included in this study from genetic data (Fig. 7). For tree construction, we assembled published sequence data of 65 lemur using Genbank (NCBI) for five mitochondrial (12S, 16S, COX2, ND3-4) and six nuclear loci (IRPB, MCR1, ABCA1, ADORA3, FGA, NRAMP). Not all species had sequence data available for all loci. Missing nucleotide data were coded as missing data (“?”) and aligned using the ClustalW algorithm in Mesquite. Alignments were checked by eye. We used MrBayes to calculate a consensus tree using a partitioned model. Substitution models were calculated using JModelTest. The MCMC algorithm was run for 10,000,000 generations with a sampling frequency of 1000 and burn-in of 25%, resulting in 7500 trees to calculate the consensus tree. Due to the lack of genetic data for some species (*Phaner, Hapalemur, Prolemur* and *Propithecus candidus*) the consensus tree was subjected to three manual changes conducted in MESQUITE 3.04 in order to create a robust and “up to date” final phylogeny for further comparative analyses. First, both *Phaner* species were placed as sister clade to all other Cheirogaleidae. *Prolemur* and *Hapalemur* were made monophyletic (excluding *Lemur catta* from this clade) and *Propithecus candidus* was manually added to the tree as no sequence data were available for this taxon. All manual changes were made in accordance with the most recent and complete phylogeny by Hererra and Dávalos^75^.

#### Statistical analyses

As climatic factors, we included the maximum temperature, the upper elevation range and the minimum rainfall for each ecoregion (Muldoon & Goodman^37^). Because variation in these climate variables also correlated with habitat (rainy or dry forest; Table S10) and activity patterns, with nocturnal species occurring on average in hotter and dryer habitats at lower elevation ranges (Table S10), we included only the three climate variables in the models below.

For each facial color trait, facial color pattern complexity (total number of different hair colors in all areas), skin pigmentation as well as hair length in each region, we determined the median from all scored pictures for each species. We then fitted phylogenetic generalized least-squares (PGLS) regressions, using a Brownian motion model to test for evolutionary relationships between facial, social and ecological variables, while controlling for phylogenetic effects^76^. Facial color traits were used as response variables. As fixed factors we included either temperature, rainfall, or elevation range, and average group size as well as the number of sympatric species at the family level (as a crude measure of the risk of heterospecific mating). We could not use the number of sympatric congeneric species to operationalize this risk because only some *Eulemur ssp*. and *Microcebus ssp*. occur in sympatry with a congener. Maximum temperature, minimum annual rainfall and upper elevation range were log transformed. Facial color complexity, average group size and number of sympatric species were square-root transformed. Phylogenetic analyses were conducted using functions in the APE, GEIGER and NMLE packages in R 3.2.2 (R Core Development Team).

### Data availability

All data needed to evaluate the conclusions in the paper are present in the Supplementary Information.

## Electronic supplementary material

Supplementary information
