# Supplementary material for: Evolution of facial color pattern complexity in lemurs
Source: Sci Rep. 2017 Nov 9;7:15181. doi: 10.1038/s41598-017-15393-7 (PMC5680244; doi:10.1038/s41598-017-15393-7)
Supplement: Supplementary file 1 — Supplementary information [file 41598_2017_15393_MOESM1_ESM.pdf]

Supplementary information

Evolution of facial color pattern complexity in lemurs

Hanitriniaina Rakotonirina*[1], Peter M. Kappeler[1,2] and Claudia Fichtel[1]

[1]Behavioral Ecology & Sociobiology Unit, German Primate Center, Göttingen, Germany.
[2]Wissenschaftskolleg zu Berlin, Wallotstr. 19, 14193 Berlin, Germany.

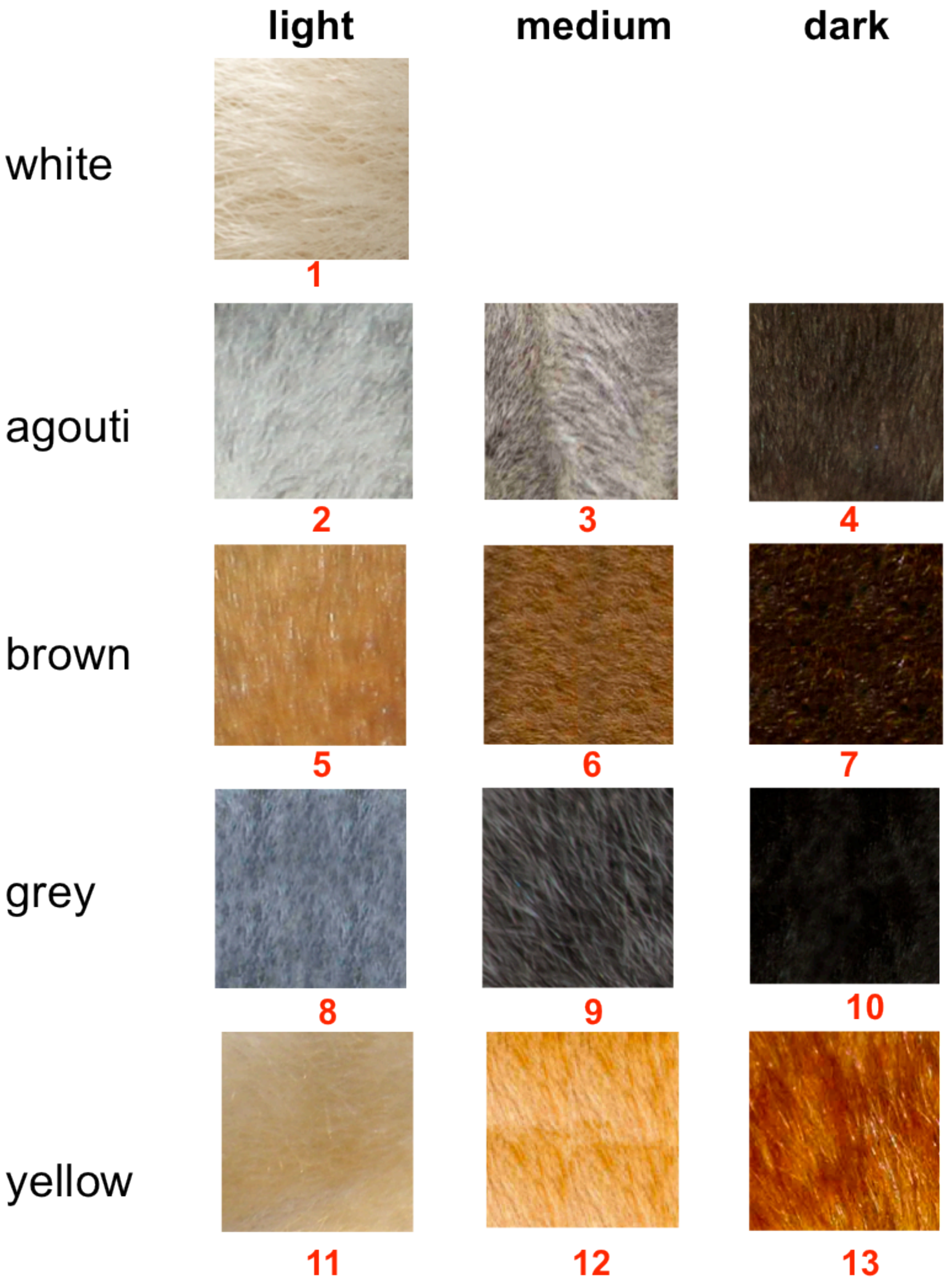

Figure S1. Categorization of hair coloration in each area of the face. 1 = white, 2 = light agouti, 3 = medium agouti, 4 = dark agouti, 5 = Light brown, 6 = medium brown, 7 = dark brown, 8 = Light grey, 9 = medium grey, 10 = black, 11 = light yellow, 12 = medium yellow and 13 = Reddish/dark yellow.

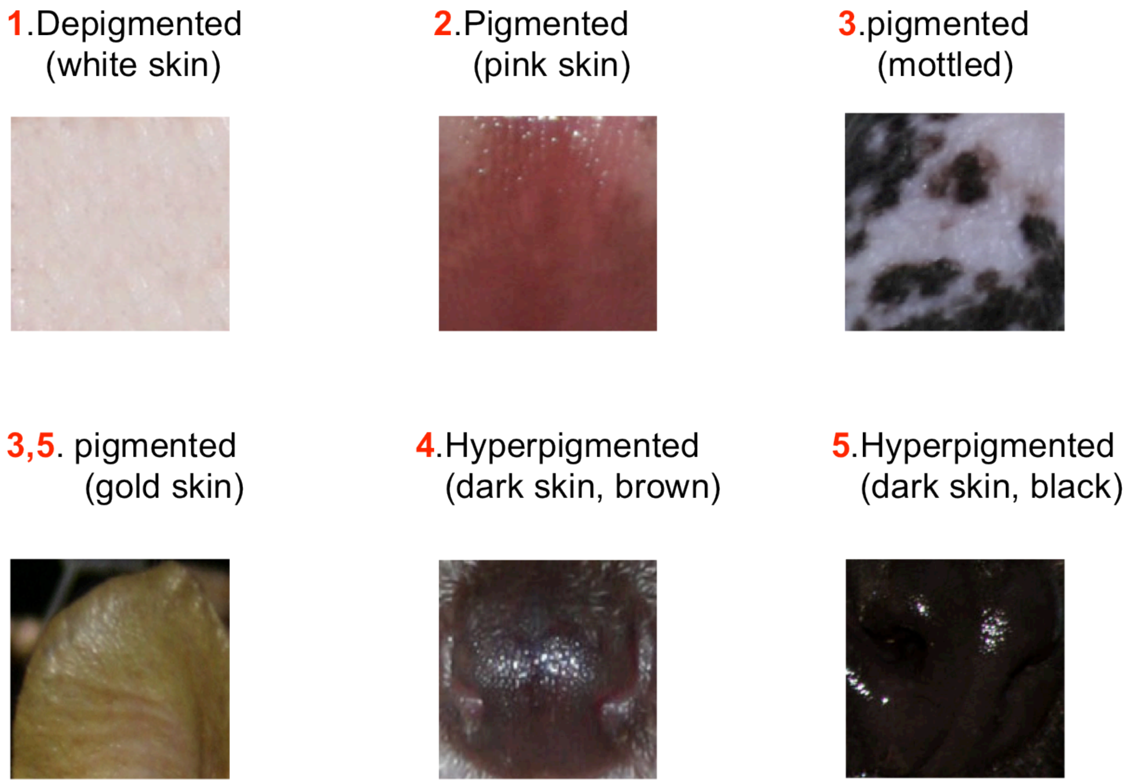

Figure S2. Categorization of skin color in the face. 1= depigmented (white skin), pigmented with 2 = pink skin, 3 = mottled, and 3,5 = gold skin (the appearance of the color is gold), hyper pigmented (dark skin) with 4 = brown, 5 = black.

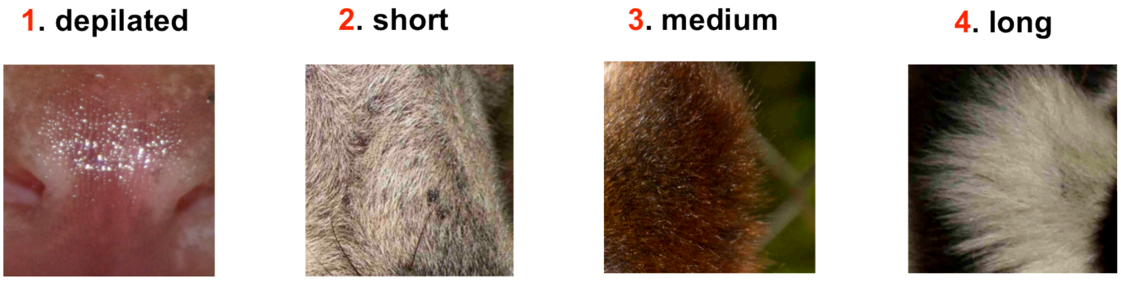

Figure S3. Categorization of hair length for each area in the face. 1 = depilated, to 2 = short hair, then 3 = Medium hair, to 4 = long hair.

Table S1. Results of the PGLS investigating social and ecological factors influencing facial color pattern complexity.

Response variables	Predictor variables	Estimate	SE	p-value
**a) Facial color complexity** λ=0.68	Intercept	0.78	0.93	0.403
	Temperature	0.45	0.27	0.103
	Number of sympatric species at family level	0.02	0.04	0.67
	Group size	-0.13	0.08	0.09
**b) Facial color complexity** λ=0.68	Intercept	2.98	0.49	<0.001
	Elevation range	-0.1	0.07	0.125
	Number of sympatric species at family level	0.01	0.04	0.717
	Group size	-0.13	0.08	0.106
**c) Facial color complexity** λ=0.7	Intercept	3.83	0.64	<0.001
	Rainfall	-0.22	0.09	**0.015**
	Number of sympatric species at family level	0.02	0.04	0.667
	Group size	-0.14	0.08	0.074

Table S2. Results of the PGLS investigating social and ecological factors influencing hair color and length on the crown.

Response variables	Predictor variables	Estimate	SE	p-value
**a) Hair color crown** λ=0	Intercept	6.71	2.96	0.027
	Temperature	-1.08	0.86	0.215
	Number of sympatric species at family level	-0.05	0.09	0.573
	Group size	-0.02	0.15	0.879
**b) Hair color crown** λ=0	Intercept	2.1	1.38	0.132
	Elevation range	0.14	0.21	0.5
	Number of sympatric species at family level	-0.04	0.09	0.663
	Group size	-0.01	0.15	0.932
**c) Hair color crown** λ=0	Intercept	0.9	1.81	0.62
	Rainfall	0.31	0.26	0.24
	Number of sympatric species at family level	-0.04	0.09	0.62
	Group size	-0.02	0.15	0.885
**d) Hair length crown** λ=0.84	Intercept	1.28	0.26	0.007
	Temperature	0.11	0.13	0.424
	Number of sympatric species at family level	0.02	0.02	0.298
	Group size	0.02	0.04	0.703

**e) Hair length crown**	Intercept	1.73	0.25	<0.001
	Elevation range	-0.01	0.03	0.705
λ=0.84	Number of sympatric species at family level	0.02	0.02	0.307
	Group size	0.02	0.04	0.722
**f) Hair length crown**	Intercept	1.84	0.34	<0.001
	Rainfall	-0.03	0.05	0.552
λ=0.84	Number of sympatric species at family level	0.02	0.02	0.299
	Group size	0.02	0.04	0.734

Table S3. Results of the PGLS investigating social and ecological factors influencing hair color and length on the forehead.

**Response variables**	**Predictor variables**	**Estimate**	**SE**	**p-value**
**a) Hair color forehead**	Intercept	9.16	3.36	0.008
	Temperature	-1.81	0.97	0.068
λ=0	Number of sympatric species at family level	-0.04	0.1	0.676
	Group size	-0.02	0.17	0.259
**b) Hair color forehead**	Intercept	1.83	1.58	0.252
	Elevation range	0.17	0.24	0.468
λ=0	Number of sympatric species at family level	-0.02	0.1	0.845
	Group size	-0.16	0.17	0.354
**c) Hair color forehead**	Intercept	-1.45	2.03	0.478
	Rainfall	0.64	0.29	**0.032**
λ=0	Number of sympatric species at family level	-0.04	0.1	0.716
	Group size	-0.21	0.17	0.224
**d) Hair length forehead**	Intercept	2.17	0.63	<0.001
	Temperature	-0.25	0.18	0.175
λ=0.66	Number of sympatric species at family level	0.03	0.03	0.276
	Group size	0.09	0.05	0.107
**e) Hair length forehead**	Intercept	1.04	0.33	0.003
	Elevation range	0.04	0.05	0.354
λ=0.64	Number of sympatric species at family level	0.03	0.03	0.262
	Group size	0.09	0.05	0.106
**f) Hair length forehead**	Intercept	0.87	0.45	0.056
	Rainfall	0.06	0.06	0.295
λ=0.65	Number of sympatric species at family level	0.03	0.02	0.275
	Group size	0.09	0.05	0.093

Table S4. Results of the PGLS investigating social and ecological factors influencing hair color and length around the eyes.

Response variables	Predictor variables	Estimate	SE	p-value
**a) Hair color**	Intercept	8.24	1.69	<0.001
around the eyes	Temperature	-1.57	0.5	**0.003**
λ=0.52	Number of sympatric species at family level	0.1	0.06	0.111
	Group size	0.08	0.13	0.524
**b) Hair color**	Intercept	1.27	0.89	0.16
around the eyes	Elevation range	0.25	0.12	0.052
λ=0.48	Number of sympatric species at family level	0.12	0.06	0.07
	Group size	0.07	0.13	0.577
**c) Hair color**	Intercept	0.12	1.15	0.918
around the eyes	Rainfall	0.4	0.16	**0.014**
λ=0.48	Number of sympatric species at family level	0.1	0.06	0.096
	Group size	0.09	0.13	0.485
**d) Hair length**	Intercept	1.34	0.17	<0.001
around the eyes	Temperature	0.01	0.04	0.847
λ=1	Number of sympatric species at family level	-0.01	0.01	0.571
	Group size	-0.01	0.03	0.615
**e) Hair length**	Intercept	1.41	0.13	<0.001
around the eyes	Elevation range	0.001	0.01	0.949
λ=1	Number of sympatric species at family level	-0.004	0.001	0.592
	Group size	-0.013	0.03	0.597
**f) Hair length**	Intercept	1.44	0.12	<0.001
around the eyes	Rainfall	-0.003	0.01	0.772
λ=1	Number of sympatric species at family level	-0.005	0.01	0.544
	Group size	-0.01	0.03	0.63

Table S5. Results of the PGLS investigating social and ecological factors influencing hair color and length of the face margins.

Response variables	Predictor variables	Estimate	SE	p-value
**a) Hair color**	Intercept	7.8	3.09	0.014
face margins	Temperature	-1.56	0.9	0.087
λ=0	Number of sympatric species at family level	-0.1	0.09	0.273
	Group size	-0.08	0.15	0.597
**b) Hair color**	Intercept	0.26	1.43	0.859
face margins	Elevation range	0.34	0.22	0.12
λ=0	Number of sympatric species at family level	-0.09	0.09	0.321
	Group size	-0.1	0.16	0.55

**c) Hair color** face margins λ=0	Intercept	-1.79	1.85	0.338
	Rainfall	0.62	0.27	**0.024**
	Number of sympatric species at family level	-0.1	0.09	0.275
	Group size	-0.1	0.15	0.508
**c) Hair length** face margins λ=1	Intercept	15.2	0.24	<0.001
	Temperature	-0.02	0.06	0.7
	Number of sympatric species at family level	0.001	0.01	0.97
	Group size	0.08	0.04	**0.039**
**d) Hair length** face margins λ=1	Intercept	1.45	0.19	<0.001
	Elevation range	-0.001	0.02	0.938
	Number of sympatric species at family level	-0.001	0.01	0.931
	Group size	0.08	0.04	**0.034**
**e) Hair length** face margins λ=1	Intercept	1.44	0.17	<0.001
	Rainfall	0.001	0.02	0.994
	Number of sympatric species at family level	-0.001	0.01	0.939
	Group size	0.08	0.04	**0.036**

Table S6. Results of the PGLS investigating social and ecological factors influencing hair color and length of the ears.

Response variables	Predictor variables	Estimate	SE	p-value
**a) Hair color** ears λ=0	Intercept	10.27	5.15	0.055
	Temperature	-1.97	1.56	0.217
	Number of sympatric species at family level	-0.04	0.17	0.82
	Group size	-0.47	0.24	0.059
**b) Hair color** ears λ=0	Intercept	0.08	3.18	0.979
	Elevation range	0.52	0.43	0.242
	Number of sympatric species at family level	-0.02	0.18	0.922
	Group size	-0.49	0.24	0.05
**c) Hair color** ears λ=0	Intercept	-2.95	4.17	0.486
	Rainfall	0.92	0.56	0.113
	Number of sympatric species at family level	-0.04	0.17	0.819
	Group size	-0.46	0.24	0.059
**d) Skin color** ears λ=0	Intercept	2.16	1.65	0.203
	Temperature	-0.1	0.48	0.841
	Number of sympatric species at family level	-0.08	0.04	0.07
	Group size	0.33	0.12	**0.009**
**e) Skin color** ears λ=0	Intercept	1.27	0.7	0.079
	Elevation range	0.09	0.1	0.416
	Number of sympatric species at family level	-0.08	0.04	0.066
	Group size	0.33	0.12	**0.01**

**f) Skin color** ears λ=0	Intercept	1.76	0.86	0.049
	Rainfall	0.01	0.12	0.942
	Number of sympatric species at family level	-0.08	0.04	0.071
	Group size	0.33	0.12	**0.01**
**g) Hair length** ears λ=0.75	Intercept	3.87	0.93	<0.001
	Temperature	-0.88	0.27	**0.002**
	Number of sympatric species at family level	0.03	0.04	0.439
	Group size	0.2	0.08	**0.019**
**h) Hair length** ears λ=0.79	Intercept	-0.37	0.51	0.463
	Elevation range	0.19	0.07	0.**007**
	Number of sympatric species at family level	0.03	0.04	0.414
	Group size	0.19	0.08	**0.028**
**i) Hair length** ears λ=0.77	Intercept	-0.44	0.71	0.541
	Rainfall	0.19	0.09	**0.049**
	Number of sympatric species at family level	0.03	0.04	0.449
	Group size	0.21	0.09	**0.02**

Table S7. Results of the PGLS investigating social and ecological factors influencing skin color of the nose.

Response variables	Predictor variables	Estimate	SE	p-value
**a) Skin color** nose λ=1	Intercept	1.55	0.34	<0.001
	Temperature	0.06	0.09	0.507
	Number of sympatric species at family level	-0.02	0.02	0.103
	Group size	0.07	0.05	0.202
**b) Skin color** nose λ=1	Intercept	1.78	0.26	<0.001
	Elevation range	-0.001	0.03	0.834
	Number of sympatric species at family level	-0.02	0.01	0.14
	Group size	0.06	0.05	0.224
**c) Skin color** nose λ=1	Intercept	1.9	0.24	<0.001
	Rainfall	-0.02	0.02	0.308
	Number of sympatric species at family level	-0.03	0.02	0.071
	Group size	0.07	0.05	0.18

Table S8. Group size and activity pattern of Lemur species included in this study.

Family	Species	Activity pattern	Average Group size	References
Cheirogaleidae	*Allocebus trichotis*	N	1,59	1;2;3
Cheirogaleidae	*Cheirogaleus crossleyi*	N	2	1;4;5
Cheirogaleidae	*Cheirogaleus major*	N	2	2;6
Cheirogaleidae	*Cheirogaleus medius*	N	2	2;7;8
Cheirogaleidae	*Cheirogaleus sibreei*	N	2	4
Cheirogaleidae	*Microcebus berthae*	N	1	9
Cheirogaleidae	*Microcebus griseorufus*	N	1	2;6
Cheirogaleidae	*Microcebus lehilahytsara*	N	1	6;10
Cheirogaleidae	*Microcebus murinus*	N	1	2;4
Cheirogaleidae	*Microcebus myoxinus*	N	1	2;6
Cheirogaleidae	*Microcebus ravelobensis*	N	1	6;11
Cheirogaleidae	*Microcebus rufus*	N	1	2;6;12;13
Cheirogaleidae	*Microcebus tavaratra*	N	1	6;13
Cheirogaleidae	*Mirza coquereli*	N	1	2;6;14
Cheirogaleidae	*Mirza zaza*	N	1	6;15
Cheirogaleidae	*Phaner furcifer*	N	2	2;6;16
Cheirogaleidae	*Phaner pallescens*	N	2,14	6;17
Daubentoniidae	*Daubentonia madagascariensis*	N	1,3	2;6;18
Indriidae	*Avahi cleesei*	N	2,95	1;6
Indriidae	*Avahi laniger*	N	2	1;2;6
Indriidae	*Avahi meridionalis*	N	2	6
Indriidae	*Avahi occidentalis*	N	3,5	1;2;6;19
Indriidae	*Avahi unicolor*	N	2	6
Indriidae	*Indri indri*	D	3,6	1;2;6;20
Indriidae	*Propithecus candidus*	D	4,33	1;2;6;21
Indriidae	*Propithecus coquereli*	D	4,5	1;2;22
Indriidae	*Propithecus coronatus*	D	3,58	1;23;24
Indriidae	*Propithecus deckenii*	D	4,07	1;24
Indriidae	*Propithecus diadema*	D	4,6	1;2;25
Indriidae	*Propithecus edwardsi*	D	5,36	1;26
Indriidae	*Propithecus perrieri*	D	3,67	1;2;27
Indriidae	*Propithecus tattersalli*	D	4,01	1;2;28;29
Indriidae	*Propithecus verreauxi*	D	5,54	2;30;31
Lemuridae	*Eulemur albifrons*	C	8,05	32
Lemuridae	*Eulemur cinereiceps*	C	6,69	32
Lemuridae	*Eulemur collaris*	C	7,03	32
Lemuridae	*Eulemur coronatus*	C	6,07	2;32
Lemuridae	*Eulemur flavifrons*	C	7,35	32
Lemuridae	*Eulemur fulvus*	C	8,46	2;32
Lemuridae	*Eulemur macaco*	C	9,56	2;32;33

Lemuridae	*Eulemur mongoz*	C	2,81	2;32;34
Lemuridae	*Eulemur rubriventer*	C	2,81	2;32
Lemuridae	*Eulemur rufifrons*	C	5,43	2;32
Lemuridae	*Eulemur rufus*	C	6,37	32
Lemuridae	*Eulemur sanfordi*	C	6,88	32
Lemuridae	*Hapalemur aloatrensis*	C	2,68	2;32
Lemuridae	*Hapalemur aureus*	C	3,14	2;32
Lemuridae	*Hapalemur griseus*	C	4,28	1;2;36;37
Lemuridae	*Hapalemur meridionalis*	C	5,08	1;38
Lemuridae	*Hapalemur occidentalis*	C	3,63	1;39
Lemuridae	*Lemur catta*	D	11,06	1;2;40
Lemuridae	*Prolemur simus*	C	9,72	1;2;37
Lemuridae	*Varecia rubra*	C	4,8	1;2;41
Lemuridae	*Varecia variegata*	C	6,03	1;2;42
Lepilemuridae	*Lepilemur ankaranensis*	N	2	2;6
Lepilemuridae	*Lepilemur dorsalis*	N	2	2;6
Lepilemuridae	*Lepilemur edwardsi*	N	2	2;6
Lepilemuridae	*Lepilemur hubbardorum*	N	2	6
Lepilemuridae	*Lepilemur leucopus*	N	2	2;6
Lepilemuridae	*Lepilemur microdon*	N	2	6
Lepilemuridae	*Lepilemur mustelinus*	N	2	2;6
Lepilemuridae	*Lepilemur randrianasoloi*	N	2	6
Lepilemuridae	*Lepilemur ruficaudatus*	N	2	43
Lepilemuridae	*Lepilemur sahamalazensis*	N	2	6
Lepilemuridae	*Lepilemur septentrionalis*	N	2	6

N: Nocturnal, C: Cathemeral, D: Diurnal

Table S9. Number of sympatric species on the family and genus level of all lemur species included in this study.

**Family**	**Species**	**Family level**	**Genus level**	**Climate PC1**
Cheirogaleidae	*Allocebus trichotis*	7	0	-1,49418108
Cheirogaleidae	*Cheirogaleus crossleyi*	16	2	-0,38142958
Cheirogaleidae	*Cheirogaleus major*	3	0	-1,49418108
Cheirogaleidae	*Cheirogaleus medius*	9	1	1,31603635
Cheirogaleidae	*Cheirogaleus sibreei*	2	1	-1,88861293
Cheirogaleidae	*Microcebus berthae*	4	1	2,05385985
Cheirogaleidae	*Microcebus griseorufus*	3	1	3,25353701
Cheirogaleidae	*Microcebus lehilahytsara*	2	0	-1,88861293
Cheirogaleidae	*Microcebus murinus*	10	5	1,31603635
Cheirogaleidae	*Microcebus myoxinus*	5	1	1,84536148
Cheirogaleidae	*Microcebus ravelobensis*	3	1	1,84536148
Cheirogaleidae	*Microcebus rufus*	7	0	-0,38142958
Cheirogaleidae	*Microcebus tavaratra*	3	1	1,84536148
Cheirogaleidae	*Mirza coquereli*	5	0	1,94961067
Cheirogaleidae	*Mirza zaza*	2	0	-0,02162573
Cheirogaleidae	*Phaner furcifer*	5	0	-1,09974922
Cheirogaleidae	*Phaner pallescens*	9	0	1,31603635
Daubentoniidae	*Daubentonia madagascariensis*	0	0	-0,38142958
Indriidae	*Avahi cleesei*	1	0	1,84536148
Indriidae	*Avahi laniger*	5	1	-1,49418108
Indriidae	*Avahi meridionalis*	1	0	-1,49418108
Indriidae	*Avahi occidentalis*	2	0	1,84536148
Indriidae	*Avahi unicolor*	0	0	-1,88861293
Indriidae	*Indri indri*	3	0	-1,49418108
Indriidae	*Propithecus candidus*	2	0	-1,88861293
Indriidae	*Propithecus coquereli*	1	0	1,84536148
Indriidae	*Propithecus coronatus*	0	0	1,84536148
Indriidae	*Propithecus deckenii*	1	0	-0,02162573
Indriidae	*Propithecus diadema*	2	0	-1,49418108
Indriidae	*Propithecus edwardsi*	2	0	-1,49418108
Indriidae	*Propithecus perrieri*	1	0	1,84536148
Indriidae	*Propithecus tattersalli*	0	0	1,84536148
Indriidae	*Propithecus verreauxi*	1	0	1,31603635
Lemuridae	*Eulemur albifrons*	4	1	-1,49418108
Lemuridae	*Eulemur cinereiceps*	6	2	-1,49418108
Lemuridae	*Eulemur collaris*	1	0	-1,49418108
Lemuridae	*Eulemur coronatus*	2	1	-0,02162573
Lemuridae	*Eulemur flavifrons*	1	0	-1,0679711
Lemuridae	*Eulemur fulvus*	6	3	-0,38142958

Lemuridae	*Eulemur macaco*	3	1	-1,88861293
Lemuridae	*Eulemur mongoz*	3	2	1,84536148
Lemuridae	*Eulemur rubriventer*	8	4	-1,49418108
Lemuridae	*Eulemur rufifrons*	7	2	0,08262346
Lemuridae	*Eulemur rufus*	4	1	1,31603635
Lemuridae	*Eulemur sanfordi*	2	1	-0,02162573
Lemuridae	*Hapalemur aloatrensis*	0	0	-1,88861293
Lemuridae	*Hapalemur aureus*	6	1	-1,88861293
Lemuridae	*Hapalemur griseus*	12	1	-1,49418108
Lemuridae	*Hapalemur meridionalis*	1	0	-1,49418108
Lemuridae	*Hapalemur occidentalis*	6	0	-0,02162573
Lemuridae	*Lemur catta*	2	0	1,31603635
Lemuridae	*Prolemur simus*	6	0	-1,88861293
Lemuridae	*Varecia rubra*	2	0	-1,09974922
Lemuridae	*Varecia variegata*	8	0	-1,49418108
Lepilemuridae	*Lepilemur ankaranensis*	0	0	1,84536148
Lepilemuridae	*Lepilemur dorsalis*	0	0	-1,88861293
Lepilemuridae	*Lepilemur edwardsi*	0	0	1,84536148
Lepilemuridae	*Lepilemur hubbardorum*	0	0	2,05385985
Lepilemuridae	*Lepilemur leucopus*	0	0	3,25353701
Lepilemuridae	*Lepilemur microdon*	0	0	-1,49418108
Lepilemuridae	*Lepilemur mustelinus*	0	0	-1,49418108
Lepilemuridae	*Lepilemur randrianasoloi*	0	0	1,84536148
Lepilemuridae	*Lepilemur ruficaudatus*	0	0	1,31603635
Lepilemuridae	*Lepilemur sahamalazensis*	0	0	1,84536148
Lepilemuridae	*Lepilemur septentrionalis*	0	0	-1,88861293

Table S10. Results of the PGLS investigating the influence of activity patterns on temperature, elevation and rainfall.

Response variables	Predictor variables	Estimate	SE	p-value
**a) Temperature**	Intercept (reference cathemeral)	3.3	0.02	<0.001
λ=0	diurnal	0.06	0.04	0.09
	nocturnal	-0.02	0.02	**0.029**
**b) Elevation**	Intercept (reference cathemeral)	7.15	0.1	<0.001
λ=0	diurnal	-0.33	0.15	**0.03**
	nocturnal	-0.37	0.12	**0.004**
**c) Rainfall**	Intercept (reference cathemeral)	7.26	0.08	<0.001
λ=0	diurnal	-0.11	0.12	0.364
	nocturnal	-0.22	0.1	**0.035**

References

1. http://www.alltheworldsprimates.org

2. Kappeler, P.M., 1997. Determinants of primate social organization: comparative evidence and new insights from Malagasy lemurs. *Biological Reviews*, *72*(1), 111-151.

3. Biebouw, K., 2009. *Revealing the behavioural ecology of the elusive hairy-eared dwarf lemur (Allocebus trichotis)* (Doctoral dissertation, Oxford Brookes University).

4. Schülke, O. and Ostner, J., 2005. Big times for dwarfs: social organization, sexual selection, and cooperation in the Cheirogaleidae. *Evolutionary Anthropology: Issues, News, and Reviews*, *14*(5), pp.170-185.

5. Blanco, M.B., Godfrey, L.R., Rakotondratsima, M., Rahalinarivo, V., Samonds, K.E., Raharison, J.L. & Irwin, M.T., 2008. Discovery of sympatric dwarf lemur species in the high-altitude rain forest of Tsinjoarivo, Eastern Madagascar: implications for biogeography and conservation. *Folia Primatologica*, 80(1),1-17.

6. Mittermeier, R. A., Louis Jr, E. E., Richardson, M., Schwitzer, C., Langrand, O., Rylands, A.B., Hawkins, F., Rajaobelina, S., Ratsimbazafy, J., Rasoloarison, R. M. & Roos, C. 2010. Lemurs of Madagascar, 3rd edn, Tropical Field Guide Series. *Conservation International, Arlington, VA*.

7. Müller, A.E., 1998. A preliminary report on the social organisation of Cheirogaleus medius (Cheirogaleidae; Primates) in north-west Madagascar. *Folia Primatologica*, 69(3), 160-166.

8. Müller, A.E., 1999. Social organization of the fat-tailed dwarf lemur (*Cheirogaleus medius*) in northwestern Madagascar. In *New directions in lemur studies* (pp. 139-157). Springer US.

9. Dammhahn, M. & Kappeler, P.M., 2005. Social system of *Microcebus berthae*, the world's smallest primate. *International Journal of Primatology*, *26*(2), 407-435.

10. Jürges, V., Kitzler, J., Zingg, R. & Radespiel, U., 2013. First insights into the social organisation of Goodman's mouse lemur (*Microcebus lehilahytsara*)–testing

predictions from socio-ecological hypotheses in the Masoala Hall of Zurich Zoo. *Folia Primatologica*, *84*(1), 32-48.

11. Braune, P., Schmidt, S. & Zimmermann, E., 2005. Spacing and group coordination in a nocturnal primate, the golden brown mouse lemur (*Microcebus ravelobensis*): the role of olfactory and acoustic signals. *Behavioral Ecology and Sociobiology*, *58*(6), 587-596.

12. Atsalis, S., 2000. Spatial distribution and population composition of the brown mouse lemur (*Microcebus rufus*) in Ranomafana National Park, Madagascar, and its implications for social organization. *American Journal of Primatology*, 51(1), 61-78.

13. Kappeler P.M. & Rasoloarison R.M., 2003. *Microcebus*, Mouse Lemurs, Tsidy. Goodman SM, Benstead JP (eds). *The Natural History of Madagascar*, 1310-1315, University of Chicago Press. Chicago.

14. Pages, E., 1980. Ethoecology of Microcebus coquereli during the dry season. *Nocturnal Malagasy primates: Ecology, physiology and behavior*, 97-116.

15. Rode, E.J., Nekaris, K.A.I., Markolf, M., Schliehe-Diecks, S., Seiler, M., Radespiel, U. & Schwitzer, C., 2013. Social organisation of the northern giant mouse lemur Mirza zaza in Sahamalaza, north western Madagascar, inferred from nest group composition and genetic relatedness. *Contributions to Zoology*, *82*(2).

16. Schülke, O., 2005. Evolution of pair-living in *Phaner furcifer*. *International Journal of Primatology*, *26*(4), 903-919.

17. Schülke, O. and Kappeler, P.M., 2003. So near and yet so far: territorial pairs but low cohesion between pair partners in a nocturnal lemur, Phaner furcifer. *Animal Behaviour*, *65*(2), 331-343.

18. Sterling, E.J., 1993. Patterns of range use and social organization in aye-ayes (*Daubentonia madagascariensis*) on Nosy Mangabe. In *Lemur social systems and their ecological basis* (pp. 1-10). Springer US.

19. Thalmann, U., 2001. Food resource characteristics in two nocturnal lemurs with different social behavior: *Avahi occidentalis* and *Lepilemur edwardsi*. *International Journal of Primatology*, *22*(2), 287-324.

20. Pollock, J.I., 1979. Female dominance in *Indri indri*. *Folia Primatologica*, *31*(1-2), 143-164.

21. Kelley, E. & Mayor, M.I., 2002. Preliminary study of the silky sifaka (*Propithecus diadema candidus*) in northeast Madagascar. *Lemur News*, *7*, 16-18.

22. Kun-Rodrigues, C., Salmona, J., Besolo, A., Rasolondraibe, E., Rabarivola, C., Marques, T.A. & Chikhi, L., 2014. New density estimates of a threatened sifaka species (*Propithecus coquereli*) in Ankarafantsika National Park. *American Journal of Primatology*, *76*(6), 515-528.

23. Pichon, C., Tarnaud, L., Bayart, F., Hladik, A., Hladik, C.M. & Simmen, B., 2010. Feeding ecology of the crowned sifaka (*Propithecus coronatus*) in a coastal dry forest in northwest Madagascar (SFUM, Antrema). *Lemur News*, *15*, 43-47.

24. Curtis, D.J., Velo, A., Raheliarisoa, E.O., Zaramody, A. & Müller, P., 1998. Surveys on *Propithecus verreauxi deckeni*, a melanistic variant, and *P. v. coronatus* in north-west Madagascar. *Oryx*, *32*(2), pp.157-164.

25. Irwin, M.T., 2008. Feeding ecology of *Propithecus diadema* in forest fragments and continuous forest. *International Journal of Primatology*, *29*(1), 95-115.

26. Lehman, S.M., Ratsimbazafy, J., Rajaonson, A. & Day, S., 2006. Ecological correlates to lemur community structure in southeast Madagascar. *International journal of primatology*, *27*(4), 1023-1040.

27. Banks, M.A., Ellis, E.R. and Wright, P.C., 2007. Global population size of a critically endangered lemur, Perrier's sifaka. *Animal Conservation*, *10*(2), 254-262.

28. Quéméré, E., Champeau, J., Besolo, A., Rasolondraibe, E., Rabarivola, C., Crouau-Roy, B. and Chikhi, L., 2010. Spatial variation in density and total size estimates in fragmented primate populations: the golden-crowned sifaka (*Propithecus tattersalli*). *American Journal of Primatology*, *72*(1), 72-80.

29. Vargas, A., Jiménez, I., Palomares, F. and Palacios, M.J., 2002. Distribution, status, and conservation needs of the golden-crowned sifaka (*Propithecus tattersalli*). *Biological Conservation*, *108*(3), 325-334.

30. Kappeler, P.M. and Fichtel, C., 2012. A 15-year perspective on the social organization and life history of sifaka in Kirindy Forest. In *Long-term field studies of primates* (pp. 101-121). Springer Berlin Heidelberg.

31. Jolly, A., Gustafson, H., Oliver, W.L.R. and O'Connor, S.M., 1982. *Propithecus verreauxi* population and ranging at Berenty, Madagascar, 1975 and 1980. *Folia Primatologica*, *39*(1-2), 124-144.

32. Kappeler, P.M. &Fichtel, C., 2015. The evolution of *Eulemur* social organization. *International Journal of Primatology*, *37*(1), pp.10-28.

33. Colquhoun, I.C., 1993. The socioecology of Eulemur macaco: a preliminary report. In *Lemur social systems and their ecological basis* (pp. 11-23). Springer US.

34. Curtis, D.J. and Zaramody, A., 1998. Group size, home range use, and seasonal variation in the ecology of Eulemur mongoz. *International Journal of Primatology*, *19*(5), pp.811-835.

35. Mutschler, T., Feistner, A.T. and Nievergelt, C.M., 1998. Preliminary field data on group size, diet and activity in the Alaotran gentle lemur *Hapalemur griseus alaotrensis*. *Folia Primatologica*, *69*(5), 325-330.

36. Mutschler, T., Nievergelt, C.M. and Feistner, A.T., 2000. Social organization of the Alaotran gentle lemur (*Hapalemur griseus alaotrensis*). *American Journal of Primatology*, *50*(1), 9-24.

37. Grassi, C., 2006. Variability in habitat, diet, and social structure of *Hapalemur griseus* in Ranomafana National Park, Madagascar. *American journal of physical anthropology*, *131*(1), 50-63.

38. Tan, C.L., 1999. Group composition, home range size, and diet of three sympatric bamboo lemur species (genus *Hapalemur*) in Ranomafana National Park, Madagascar. *International Journal of Primatology*, 20(4), 547-566.

39. Eppley, T.M., Verjans, E. and Donati, G., 2011. Coping with low-quality diets: a first account of the feeding ecology of the southern gentle lemur, *Hapalemur meridionalis*, in the Mandena littoral forest, southeast Madagascar. *Primates*, *52*(1), 7-13.

40. Curtis, D.J., Zaramody, A. and Rabetsimialona, O.D., 1995. Sighting of the western gentle lemur *Hapalemur griseus occidentalis* in north-west Madagascar. *Oryx*, *29*(03), 215-217.

41. Koyama, N., Nakamichi, M., Ichino, S. and Takahata, Y., 2002. Population and social dynamics changes in ring-tailed lemur troops at Berenty, Madagascar between 1989–1999. *Primates*, *43*(4), 291-314.

42. Rigamonti, M.M., 1993. Home range and diet in red ruffed lemurs (*Varecia variegata rubra*) on the Masoala Peninsula, Madagascar. In *Lemur social systems and their ecological basis* (pp. 25-39). Springer US.

43. Hilgartner, R., Zinner, D., Fichtel, C. and Kappeler, P.M., 2006. Why males live with only one female: pair-living in a nocturnal lemur (*Lepilemur ruficaudatus*). *Living apart together: Pair-living in red-tailed sportive lemurs (Lepilemur ruficaudatus)*, 74.
